# Outcome of severely injured patients in a unique trauma system with 24/7 double trauma surgeon on-call service

**DOI:** 10.1186/s13049-023-01122-9

**Published:** 2023-10-25

**Authors:** Karlijn J. P. van Wessem, Luke P. H. Leenen, R. Marijn Houwert, Kim E. M. Benders, Roger K. J. Simmermacher, Mark C. P. M. van Baal, Ivar G. J. M. de Bruin, Mirjam B. de Jong, Stefaan J. B. Nijs, Falco Hietbrink

**Affiliations:** Department of Trauma Surgery, University Medical Center Utrecht, Heidelberglaan 100, 3584 CX Utrecht, The Netherlands

**Keywords:** Polytrauma, Mortality, Double trauma surgeon on-call service

## Abstract

**Background:**

The presence of in-house attending trauma surgeons has improved efficiency of processes in the treatment of polytrauma patients. However, literature remains equivocal regarding the influence of the presence of in-house attendings on mortality. In our hospital there is a double trauma surgeon on-call system. In this system an in-house trauma surgeon is 24/7 backed up by a second trauma surgeon to assist with urgent surgery or multiple casualties. The aim of this study was to evaluate outcome in severely injured patients in this unique trauma system.

**Methods:**

From 2014 to 2021, a prospective population-based cohort consisting of consecutive polytrauma patients aged ≥ 15 years requiring both urgent surgery (≤ 24h) and admission to Intensive Care Unit (ICU) was investigated. Demographics, treatment, outcome parameters and pre- and in-hospital transfer times were analyzed.

**Results:**

Three hundred thirteen patients with a median age of 44 years (71% male), and median Injury Severity Score (ISS) of 33 were included. Mortality rate was 19% (68% due to traumatic brain injury). All patients stayed ≤ 32 min in ED before transport to either CT or OR. Fifty-one percent of patients who needed damage control surgery (DCS) had a more deranged physiology, needed more blood products, were more quickly in OR with shorter time in OR, than patients with early definitive care (EDC). There was no difference in mortality rate between DCS and EDC patients. Fifty-six percent of patients had surgery during off-hours. There was no difference in outcome between patients who had surgery during daytime and during off-hours. Death could possibly have been prevented in 1 exsanguinating patient (1.7%).

**Conclusion:**

In this cohort of severely injured patients in need of urgent surgery and ICU support it was demonstrated that surgical decision making was swift and accurate with low preventable death rates. 24/7 Physical presence of a dedicated trauma team has likely contributed to these good outcomes.

**Supplementary Information:**

The online version contains supplementary material available at 10.1186/s13049-023-01122-9.

## Background

Advances in trauma care in the last decades are likely caused by a combination of improvement in prehospital treatment, hemostatic resuscitation, and damage control surgery. Another important factor in this improvement has been the timely involvement of dedicated trauma surgeons. Several studies have examined the effect of in-house attending surgeons on process-and patient-related outcomes [1–4]. However, literature remains equivocal regarding the influence of the 24/7 presence of in-house surgeons on mortality. Some authors did not demonstrate any reduction on mortality [1, 2], whereas others provided arguments for a decrease in preventable deaths [3]. A recent review on this topic suggested that a 24/7 in-house trauma surgeon was associated with reduced mortality for severely injured patients in a level-1 trauma center setting [5].

Since 2013, there is a double trauma surgeon on-call system with 24/7 availability in our Level-1 trauma center. When the trauma team is activated, an in-house trauma surgeon is always present upon arrival of the patient in the Emergency Department (ED). Additionally, there is a second trauma surgeon available on-call to perform or assist with surgical procedures, lead resuscitation in ED if a new victim presents, or multiple victims arrive simultaneously. This schedule not only ensures that resuscitation and decision making is supervised by an experienced trauma surgeon even in multiple victims, but also that urgent surgery of often complex injuries can be performed by two trauma surgeons.

The aim of this study was to investigate outcomes by evaluating the timing and accuracy of surgical decision making in the treatment of severely injured patients within our unique trauma system.

## Methods

### Study setting

From January 2014 through December 2021, a prospective population-based cohort study was undertaken including all consecutive trauma patients ≥ 15 years who needed urgent surgery (defined as surgery ≤ 24h), and who were admitted to the Intensive Care Unit (ICU) of the University Medical Center Utrecht (UMCU). This major trauma center is the only Level-1 trauma center in the province of Utrecht and covers the central region of the Netherlands with a relatively small, but densely populated service area of 1,500 square kilometers and approximately 1.3 million residents. Around 1400 trauma patients with activation of a trauma team are annually admitted, 49% of whom arrive after hours [6]. Approximately 375 of them are multiply injured (ISS > 15) [7]. Patients with isolated injury to the brain (Abbreviated Injury Scale (AIS) head 3 or more and AIS 2 or less in other regions), asphyxiation, drowning and burns were excluded, because of possible different physiologic response to severe trauma, and a significantly different mortality and morbidity profile [8, 9].

There are no emergency physicians in our Level-1 trauma center, all patients in ED are cared for by specialty specific specialists. There is a double trauma surgeon on-call system with 24/7 availability. In case of trauma team activation, the in-house trauma surgeon is physically present upon presentation of the patient in ED. Additionally, a second trauma surgeon is available on-call (who is out of hospital during off hours, but at a 20 min response time) to perform or assist with surgical procedures or lead resuscitation in ED if multiple victims arrive simultaneously [3]. In this way high quality trauma care is continuously guaranteed. Additionally, trauma surgeons are also 24/7 available for the entire hospital for other surgical emergencies such as cricothyroidotomies. All trauma surgeons staffing Level-1 Trauma centers in the Netherlands treat both visceral and orthopedic trauma.

During the study period the CT-scanner was located in the radiology department (on a different floor in the hospital).

### Data collection

All data were prospectively collected on arrival in ED, and on a daily basis in ICU by authors KW and LL. Patient demographics, Injury Severity Score (ISS), shock and resuscitation parameters were calculated. Admission arterial blood gas analysis, coagulation status and temperature measurement were performed during resuscitation in ED as part of standard procedure. Arterial blood gas analysis and temperature measurement were repeated on arrival in ICU. Crystalloids and blood product (Packed Red Blood Cells (PRBC), Fresh Frozen Plasma (FFP) and Platelets (PLT)) use was recorded in the first 24 h following admission. Both pre-hospital and in-hospital transfer times were measured.

All traumatic deaths were weekly evaluated by the whole group of trauma surgeons according to a preset format. Charts were reviewed, including a checklist prompting specific issues. Sections of this form include resource utilization, critical time intervals, and cause of death, in addition to judgments on preventability and care. Preventability determination was based on criteria generally comparable with those propagated by the American College of Surgeons [10].

In order to evaluate quality of care a standardized mortality ratio (SMR) was calculated on an annual basis by the Dutch National Trauma Registry (DNTR) [11, 12]. SMR is the ratio between the observed in-hospital deaths and the expected numbers of deaths. The expected in-hospital mortality is calculated based on Trauma Injury Severity Score (TRISS) variables [13]. A SMR of 1 indicates that the observed mortality is similar to the expected mortality. A SMR lower than 1 suggests that the observed mortality is lower than the expected mortality, and vice versa if SMR is greater than 1, the observed mortality is higher than the expected mortality. The results are presented in a funnel plot in which each dot represents a hospital. Funnel plots visualize the relationship between sample size and precision since the control limits and distribution become more narrow with high volumes. The control limits for the funnel plots were set at 95% and 99.8% prediction intervals respectively. In case of missing data, normal (healthy) values were used. This was chosen to increase the quality of data and avoid the presentation of overly optimistic results by punishing those who had missing data [14].

### Definitions

Pre-hospital transport time was defined as time from emergency call to dispatch of the ambulance service to arrival in ED of the hospital. In-hospital times measured were: time from ED to CT-scan, time from ED to operating room (OR), time from ED to ICU. Time from ED to CT scan was defined as time of arrival in ED (including all interventions in ED) to time of first CT-scan (time used was time stamp on first CT image). Time from ED or CT to OR was calculated by using first measurement in OR which is routinely recorded in the electronic patient file. OR time was calculated from arrival in OR until departure including all peri-operative anesthestic procedures. Time from ED, CT or OR to ICU was calculated by time of arrival in ICU which is also routinely recorded in the electronic patient file.

Damage control surgery (DCS) was defined as any surgery (both truncal and orthopedic) that was abbreviated to restore normal physiology before returning to OR for definitive treatment.

In our hospital, the selection for damage control surgery is in correlation with the general literature consensus [15, 16], and based on a combination of physiological parameters (acidosis (base deficit ≤ 6.0 mEq/L), hypothermia (temperature ≤ 34 °C), coagulopathy (Prothrombin Time (PT) ≥ 16 s), anatomical locations of the injuries, associated injuries, patient’s response to the given care, and surgeon’s discretion. Patients who initially underwent DCS often needed additional surgeries during their hospital stay (fracture fixation, repeated debridement for soft tissue injuries, mesh approximation in open abdomen etc.). Early definitive care (EDC) was defined as definitive fixation of fractures, and/or definitive treatment of injuries in brain, chest and abdomen in the early phase after injury (≤ 24 h). Patients who had definitive surgery in multiple procedures stretched over several days were also included in EDC group.

Further, both Denver Multiple Organ Failure (MOF) scores [17], and ARDS Berlin criteria [18] were registered daily up until 28 days or discharge from ICU. Denver MOF score was chosen over other MOF scoring systems like Marshall Multiple Organ Dysfunction Syndrome (MODS) or Sequential Organ Failure Assessment (SOFA) to avoid difficulties by including the Glasgow Coma Scale (GCS) in the organ failure score. GCS can be challenging to obtain in trauma patients in ICU, because they are often sedated and intubated for extended periods. This could negatively influence the Central Nervous System (CNS) organ failure score [8, 9].

Primary outcome was mortality and its relation to timing and accuracy of process related decisions.

### Ethical approval

The local ethics committee approved this prospective observational study (reference number WAG/mb/16/026664).

### Statistical analysis

Data were analyzed using IBM SPSS Statistics, version 25.0 (Armonk, NY, USA). Graphs were prepared with both IBM SPSS Statistics, version 25.0 (Armonk, NY, USA), and GraphPad Prism version 9.3.0 (San Diego, CA, USA). Results are presented as median and interquartile range (IQR). Comparison of variables was done using Kruskal–Wallis test. Significant differences for categorical variables were calculated through Chi-Square test or Fisher’s exact test depending on the size of the groups. Statistical significance was defined as *P* < 0.05.

## Results

**Fig. 1:**
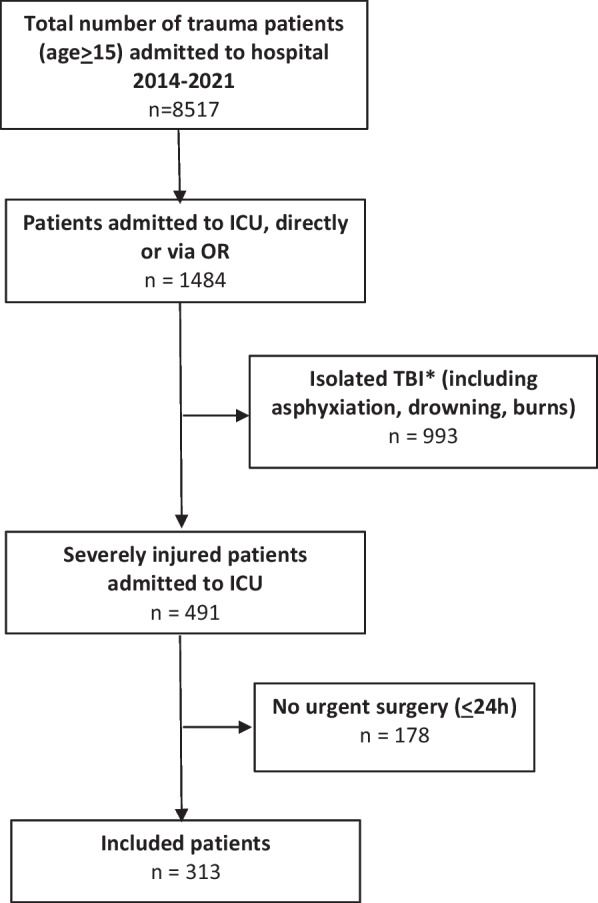
Flowchart of included patients. *Isolated traumatic brain injury (TBI) was defined as Abbreviated Injury Score (AIS) head ≥ 3 and AIS ≤ 2 or less in other regions

In this study, 313 severely injured patients (71% male) who underwent both urgent surgery and were admitted to ICU were included (Fig. 1). The median age was 44 (27–59) years, 91% of injuries were caused by a blunt mechanism with a median Injury Severity Score (ISS) of 33 (24–38). Data on physiology in both ED and ICU, crystalloid and blood product resuscitation, and outcome are presented in Table 1.

**Table 1:** Demographic and resuscitation parameters in polytrauma patients who had urgent surgery and were admitted to ICU

	Total population (n = 313)	Mortality (n = 59)	Survival (n = 254)	*P*-value
Age (years)	44 (27–59)	56 (40–73)	39 (25–56)	< 0.001*
Male gender	221 (71)	42 (71)	179 (71)	1.0
Blunt MOI	285 (91)	56 (95)	229 (90)	0.32
ISS	33 (24–38)	38 (29–48)	29 (22–38)	< 0.001*
AIS head	3 (0–4)	4 (2–5)	3 (0–4)	0.004*
AIS face	0 (0–1)	0 (0–2)	0 (0–1)	0.34
AIS chest	3 (2–4)	3 (3–4)	3 (2–4)	0.002*
AIS abdomen	2 (0–3)	2 (0–4)	2 (0–2)	0.09
AIS pelvis/extremities	3 (1–3)	3 (2–3)	3 (0–3)	0.29
AIS external	0 (0–1)	0 (0–1)	0 (0–1)	0.55
SBP_ED (mmHg)	114 (90–133)	111 (74–140)	115 (91–132)	0.29
SBP ≤ 90 mmHg_ED	84 (27)	22 (37)	62 (24)	0.03*
Hb_ED (mEq/L)	7.9 (7.0–8.9)	7.6 (6.1–8.4)	8.0 (7.2–8.9)	0.004*
pH_ED	7.31 (7.24–7.37)	7.25 (7.10–7.34)	7.32 (7.26–7.37)	< 0.001*
PaC02_ED (mmHg)	45 (40–52)	48 (43–57)	44 (39–51)	0.003*
BD _ED (mEql/L)	−4.0 (−7.0–1.0)	−7.0 (−10.1–3.0)	−3.0 (−6.0–1.0)	< 0.001*
PT_ED (sec)	14.7 (13.3–17.4)	15.6 (14.3–21.1)	14.3 (13.1–16.8)	< 0.001*
Temperature_ED (^o^C)	35.4 (34.5–36.4)	35.1 (34.1–35.9)	35.5 (34.5–36.5)	0.17
SBP_ICU (mmHg)	117 (101–135)	115 (95–135)	118 (104–135)	0.15
Hb_ICU (mmol/L)	7.4 (6.6–8.1)	7.3 (6.2–7.8)	7.4 (6.7–8.2)	0.09
pH_ICU	7.34 (7.28–7.38)	7.30 (7.23–7.34)	7.34 (7.29–7.38)	< 0.001*
PaCO2_ICU (mmHg)	41 (36–46)	43 (38–50)	41 (36–46)	0.048*
BD_ICU (mEq/L)	−4.1 (−6.6–2.1)	−5.5 (−10.0–3.4)	−4.0 (−5.9–1.9)	< 0.001*
Temperature_ICU (^o^C)	35.1 (34.4–35.9)	35.1 (34.1–35.7)	35.2 (34.4–36.0)	0.15
UO_ICU (ml)	145 (80–300)	165 (80–363)	140 (80–300)	0.32
Resuscitation parameters				
Crystalloids ≤ 8h (L)	5.3 (3.9–7.2)	6.2 (3.9–6.7)	5.2 (3.9–6.7)	0.003*
PRBC ≤ 8h (U)	3 (0–7)	5 (2–13)	3 (0–6)	0.003*
FFP ≤ 8h (U)	3 (0–6)	4 (1–10)	2 (0–6)	0.003*
PLT ≤ 8h (U)^#^	0 (0–1)	1 (0–2)	0 (0–1)	0.002*
Crystalloids ≤ 24h (L)	8.6 (6.7–11.2)	11.3 (8.0–13.6)	8.3 (6.6–10.6)	< 0.001*
PRBC ≤ 24h (U)	4 (0–8)	5 (1–13)	3 (0–7)	0.046*
PRBC ≥ 10 units ≤ 24h	57 (18)	19 (32)	38 (15)	0.002*
FFP ≤ 24h (U)	4 (0–8)	5 (0–14)	3 (0–8)	0.03*
PLT ≤ 24h (U)^#^	0 (0–2)	1 (0–3)	0 (0–1)	0.03*
TXA ≤ 24h	244 (78)	46 (78)	198 (78)	1.0
Outcome parameters				
Ventilator days	6 (2–11)	6 (2–13)	6 (2–11)	0.69
ICU LOS (days)	7 (3–13)	6 (2–13)	7 (3–14)	0.24
H-LOS (days)	22 (12–34)	7 (2–13)	25 (17–36)	< 0.001*
MODS	51 (16)	18 (31)	33 (13)	0.003*
ARDS	12 (4)	2 (3)	10 (4)	1.0
Infectious complications	140 (45)	17 (29)	123 (48)	0.006*
Thrombo-embolic complications	34 (11)	4 (7)	30 (12)	0.36

Data are expressed in median (IQR) or absolute numbers (%)

*MOI* mechanism of injury, *ISS* injury severity score, *AIS* abbreviated injury scale, *ED* emergency department, *SBP* systolic blood pressure, *Hb* hemoglobin, *PaC02* partial pressure of carbon dioxide in arterial blood, *BD* base deficit, *PT* prothrombin time, *UO* urinary output first hr in ICU, *PRBC* packed red blood cells, *FFP* fresh frozen plasma, *PLT* platelets, *TXA* tranexamic acid, *ICU* intensive care unit, *LOS* length of stay, *H-LOS* hospital length of stay, *MODS* multiple organ dysfunction syndrome, *ARDS* adult respiratory distress syndrome

*Statistically significant

^#^1 unit of platelets contains 5 donors

### Patient’s physiology and its relation to transport time/order and mortality

**Fig. 2:**
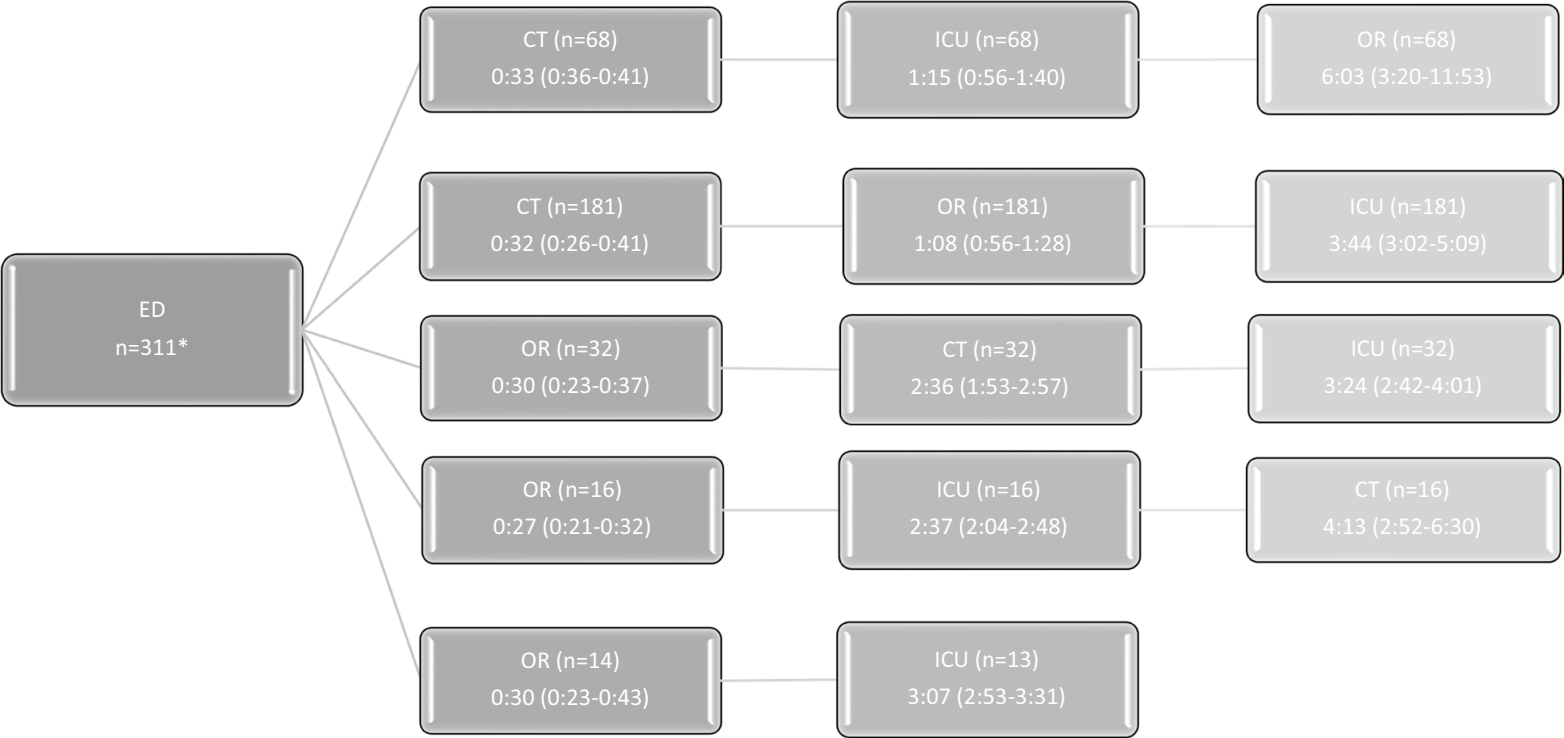
Transport order and transport times. *1 patient went from ED to ICU and subsequently OR, another patient from ED to ICU, OR, and CT. ED = emergency department, OR = operating room, ICU = intensive care unit. Transport times are expressed in h:mm (IQR)

Prehospital transport time was 1 h (0:55–1:10). Sixty-two patients (20%) went straight from ED to OR with a median time from presentation to start surgery of 29 (23–38) min. All other patients had CT prior to OR. Patients who had CT after ED needed a median time of 32 (26–42) min. Based on transport order patients were divided into five categories. Transfer times are shown per category in Fig. 2.

Fifty-one percent (n = 161) of patients needed damage control surgery (DCS) during the first surgical procedure, all others had early definitive care (EDC). Patients who underwent DCS were younger, had a more deranged physiology, and received more blood products than EDC patients. However, there was no difference in hemoglobin levels during surgery between both groups, and they received similar amounts of crystalloids during surgery. DCS and EDC patients had comparable ISS, although there was a difference in type of injuries; DCS patients were more severely injured to abdomen and pelvis/extremities, whereas EDC patients were more severely injured to brain and chest (Table 2). DCS patients went more often to OR prior to CT than patients who received EDC (31%, (50/161) vs. 9% (14/152), *p* < 0.001). DCS patients also went more rapidly from ED to OR, and stayed shorter in OR than EDC patients (Table 2). there was no difference in both prehospital to ED time (1:01 (0:56–1:10 vs. 1:00 (0:55–1:10), *p* = 0.78), and time from ED to ICU (3:16 (2:39–4:05) vs. 2:55 (1:26–4:18), *p* = 0.21) between DCS and EDC patients.

**Table 2:** Physiology and duration of surgery related to damage control surgery (DCS) and early definite care (EDC)

	DCS (n = 161)	EDC (n = 152)	*P*-value
Age (years)	35 (24–54)	49 (32–66)	< 0.001*
ISS	34 (23–41)	33 (25–38)	0.84
AIS head	3 (0–3)	3 (2–5)	< 0.001*
AIS face	0 (0–1)	0 (0–2)	0.20
AIS chest	3 (1–4)	3 (3–4)	0.006*
AIS abdomen	3 (1–4)	0 (0–2)	< 0.001*
AIS pelvis/extremities	3 (2–3)	2 (0–3)	< 0.001*
AIS external	0 (0–1)	1 (0–1)	0.74
Time from ED to OR (h:mm)	1:00 (0:37–1:19)	1:36 (0:56–5:21)	< 0.001*
OR Duration (h:mm)	1:50 (1:20–2:35)	2:10 (1:30–3:28)	0.007*
BD_OR (mEq/L)	−7.0 (−10.0–4.0)	−4.0 (−7.0–1.3)	< 0.001*
Hb_OR (mmol/L)	6.8 (5.6–7.6)	6.8 (5.9–7.8)	0.26
Temperature_OR (^o^C)	34.8 (33.8–35.4)	35.1 (34.4–36.0)	< 0.001*
Crystalloids_OR (L)	3.0 (2.0–5.0)	3.0 (2.0–5.0)	0.34
PRBC_OR (U)	3 (1–7)	0 (0–2)	< 0.001*
FFP_OR (U)	4 (1–7)	0 (0–2)	< 0.001*
PLT_OR (U)^#^	0 (0–1)	0 (0–0)	< 0.001*

Data are expressed in median (IQR), * statistically significant

^#^ 1 unit of platelets contains 5 donors

ISS = injury severity score, AIS = abbreviated injury scale, ED = Emergency Department, OR = operating room, Hb = hemoglobin, BD = Base Deficit, PRBC = packed red blood cells, FFP = fresh frozen plasma, PLT = platelets

Nineteen percent (n = 59) of patients died, the vast majority due to TBI (68%). Other causes of death included hemorrhage (8%), respiratory insufficiency (7%), ischemia after entrapment of (a major part of) the body (5%), cardiac origin (5%), MODS (3%), hypoxia and sepsis (both 2%, Fig. 4A).

Patents who later died were older, and more severely injured. Further, they had a more deranged physiology in ED, received more crystalloids and blood products both ≤ 8h and ≤ 24h, and remained more acidotic on arrival in ICU (Table 1). Although deceased patients developed more often MODS, there was no difference in ventilator days, nor in days in ICU compared to surviving patients (Table 1). Patients who later died, with most severe injuries located to head and chest, underwent more often a craniotomy, whereas survivors needed more external fixators and fractures fixations. Surgical procedures in patients who survived and who died are shown in Table 3. All 5 patients who later died of hemorrhage had an urgent laparotomy, and all 14 patients who had a craniotomy and later died, died of TBI.

**Table 3:** Type of surgery during first session in OR related to mortality

Surgical procedure*	Mortality (n = 59)	Survival (n = 254)	Total
Thoracotomy	4 (6)	12 (4)	16 (4)
Laparotomy	24 (33)	90 (27)	114 (28)
Craniotomy	14 (19)	18 (5)	32 (8)
Spine fixation	3 (4)	31 (9)	34 (8)
Fracture fixation	2 (3)	40 (12)	42 (10)
External fixator extremities/pelvis	12 (17)	92 (27)	104 (25)
Vascular procedure	2 (3)	23 (7)	25 (6)
Miscellaneous^#^	11 (15)	32 (9)	43 (10)
Total *	72	338	410

Data are expressed as absolute numbers (%)

^*^Several patients had more than one procedure during their session in OR

^#^Miscellaneous procedures included insertion of intracranial pressure (ICP) monitor, extraventricular drain, haloframe, amputation extremity, fasciotomy, debridement of soft tissue injuries, neck exploration

Twenty seven percent of patients (16/59) who later died went directly from ED to OR, all others had a CT-scan prior to surgery. Nine patients died within 24h after trauma (5 due to hemorrhage, 2 due to TBI, 1 due to ischemia, 1 due to cardiac injury), 8 of them went straight from ED to OR. One other patient with severe chest (AIS chest 3) and abdominal injuries (AIS abdomen 4) who later died due to hemorrhage went to CT prior to OR. Possibly, death could have been prevented if the patient was directly transported from ED to OR (preventable death rate 1.7%). There was no difference in transport order nor in transport times between patients who later died and the ones who survived.

**Fig. 3:**
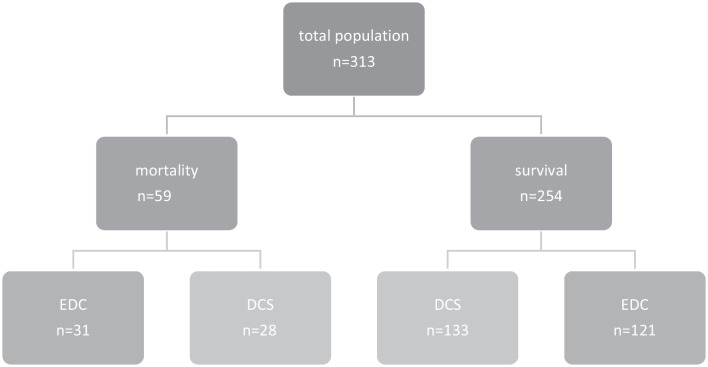
Number of patients in relation to mortality and damage control surgery. EDC = early definitive care, DCS = damage control surgery

Seventeen percent of all DCS patients (28/161) died, and vice versa 47% (28/59) of deceased patients underwent damage control surgery (Fig. 3). DCS patients who later died were older, more severely injured, mainly located in chest and abdomen. Further, they were more acidotic, had lower temperatures, and received more blood products than DCS patients who survived (Table 4). DCS patients who later died needed more often a laparotomy, whereas survivors had more often external fixators. There was no difference in type of DCS surgery between patients who later died and the survivors (*p* = 0.34, Table 4).

**Table 4:** Physiology and duration of damage control surgery (DCS) in patients who died and who survived

OR-1 DCS	Mortality (n = 28)	Survival (n = 133)	*P*-value
Age (years)	52 (32–72)	34 (24–52)	< 0.001*
ISS	41 (30–50)	29 (22–38)	0.006*
AIS head	3 (0–4)	2 (0–3)	0.27
AIS face	0 (0–1)	0 (0–1)	0.69
AIS chest	4 (3–4)	3 (0–3)	0.002*
AIS abdomen	4 (2–4)	3 (0–4)	0.003*
AIS pelvis/extremities	3 (2–3)	3 (2–3)	0.59
AIS external	0 (0–1)	0 (0–1)	0.47
Time from ED to OR (h:mm)	0:57 (0:28–1:29)	1:00 (0:39–1:17)	0.58
Duration of surgery (h:mm)	2:00 (1:21–2:52)	1:50 (1:20–2:35)	0.77
BD_OR (mEq/L)	−12.0 (–20.5–7.0)	−6.0 (−9.0–3.1)	< 0.001*
Hb_OR (mmol/L)	6.4 (5.2–7.2)	6.8 (5.7–7.6)	0.15
Temperature_OR (°C)	33.7 (32.9–35.3)	34.8 (34.0–35.4)	0.04*
Crystalloids_OR (L)	3.0 (2.0–3.8)	3.0 (2.0–5.0)	0.28
PRBC_OR (U)	8 (3–15)	3 (1–5)	< 0.001*
FFP_OR (U)	8 (4–15)	3 (0–6)	< 0.001*
PLT_OR (U)^#^	2 (0–3)	0 (0–1)	< 0.001*
Surgical procedure**			Total
Thoracotomy	3 (8)	4 (2)	7 (3)
Laparotomy	21 (54)	70 (34)	91 (37)
Craniotomy	2 (5)	3 (1)	5 (2)
Spine fixation	0	4 (2)	4 (2)
Fracture fixation	0	9 (4)	9 (4)
External fixator extremities/pelvis	12 (31)	92 (45)	104 (42)
Vascular procedure	1 (3)	9 (4)	10 (4)
Miscellaneous^§^	0	15 (7)	15 (6)
Total	39	206	245

Data are expressed in median (IQR) or absolute numbers (%), * statistically significant

*ISS* injury severity score, *AIS* abbreviated injury scale, *ED* emergency department, *OR* operating room, *Hb* hemoglobin, *BD* base deficit, *PRBC* packed red blood cells, *FFP* fresh frozen plasma, *PLT* platelets

^#^1 unit of platelets contains 5 donors

**Several patients had more than one surgical procedure

^§^Miscellaneous procedures included insertion of ICP meter, extraventricular drain, haloframe, amputation extremity, fasciotomy, debridement of soft tissue injuries, neck exploration

### Physiology in patients who later died

All DCS patients who later died had a more deranged physiology and needed more blood products than deceased EDC patients (Additional file 2: Table S1).

**Fig. 4:**
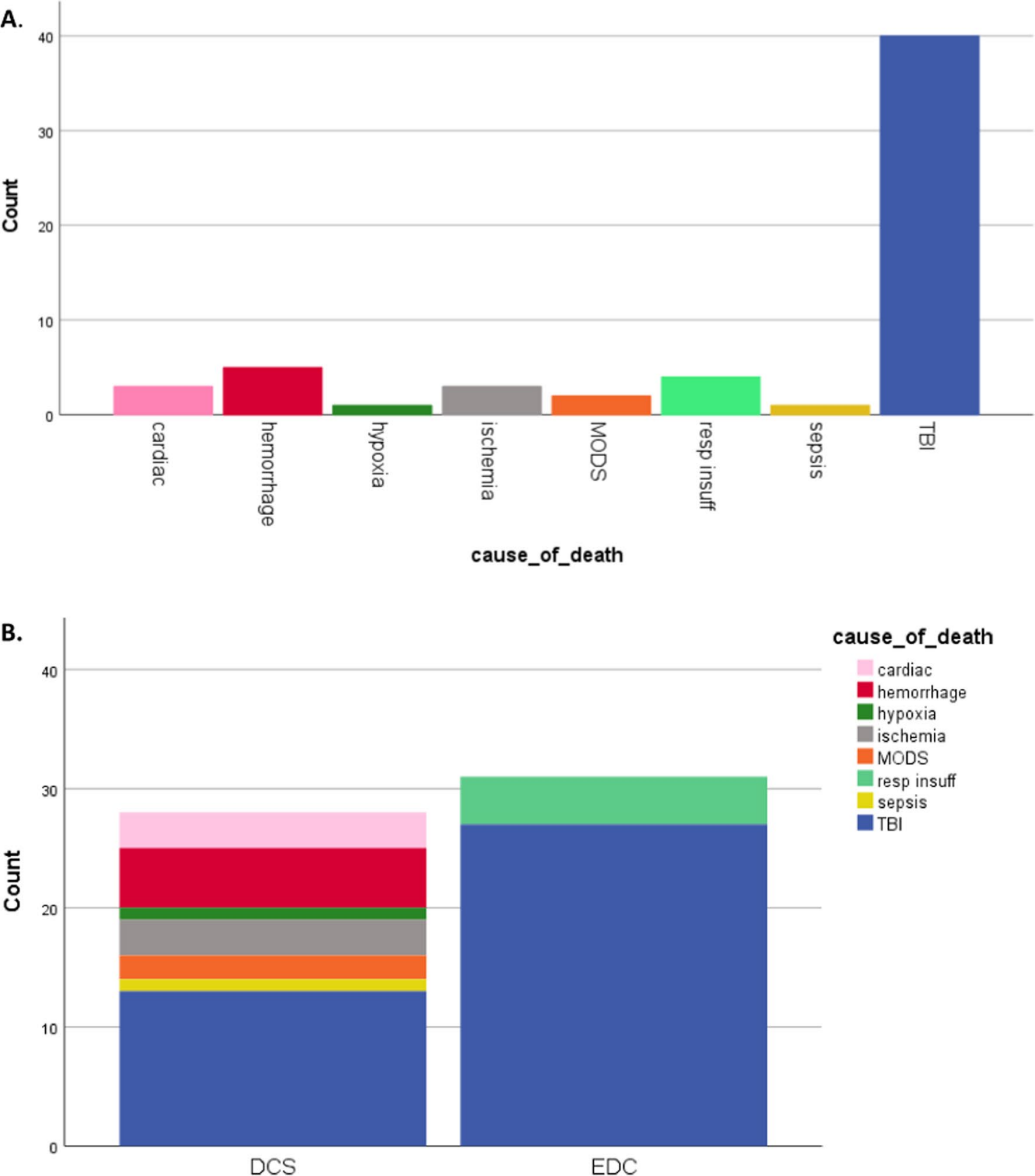
**A** Cause of death in studied population. **B** Cause of death related to damage control surgery (DCS) and early definitive care (EDC)

Although there was no difference in mortality rate between patients with DCS compared to EDC (17% (28/161) vs. 20% (31/152), *p* = 0.50), there was a difference in cause of death; Eighty-seven percent of EDC patients died of TBI, and 13% of respiratory insufficiency, whereas the cause of death in DCS patients was more diverse; Forty-six percent (13/28) died of TBI, 18% (5/28) died of hemorrhage, 11% (3/28) of ischemia by entrapment, 11% (3/28) of cardiac origin, 7% (2/28) of MODS, and 4% (1/28) of hypoxia and sepsis (*P* < 0.001, Fig. 4B). There was also a difference in time of death; DCS patients died 5 (1–13) days after admission compared to 9 (3–16) days in EDC patients (*P* < 0.001).

### Outcome related to time of surgery

**Fig. 5:**
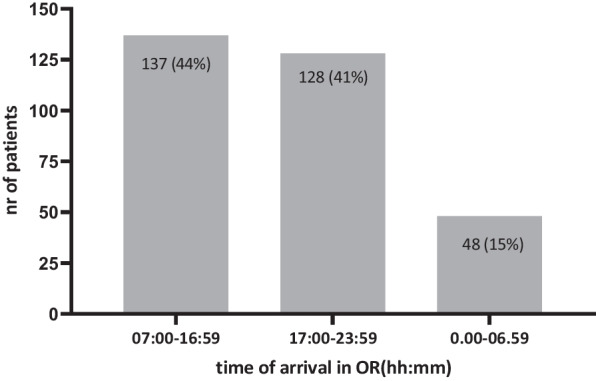
Time of arrival in OR. Data are expressed in absolute numbers (%)

Fifty-six percent of patients (176/313) had surgery during off-hours (Fig. 5). There was no difference in demographics nor in physiology in ED between patients who had surgery during daytime and patients who needed urgent surgery during evening and night time. Additionally, there was no difference in time from ED to OR, type of surgery, nor duration of surgery. There was also no difference in outcome between patients who surgery during daytime and off-hours (Table 5).

**Table 5:** Demographics, physiology, and outcome in patients who had daytime surgery (07:00–16:59) and who had surgery during off-hours (17:00–06:59)

	Day time surgery (n = 137)	Off-hours surgery (n = 176)	*P*-value
Age (years)	47(29–61)	40 (25–57)	0.25
Blunt MOI	125 (91)	160 (91)	0.92
ISS	29 (22–40)	34 (26–38)	0.28
SBP_ED (mmHg)	110 (85–134)	115 (94–132)	0.77
BD_ED (mEq/L)	−4.0 (–7.5–1.0)	−3.0 (−7.0–1.0)	0.77
Hb_ED (mmol/L)	7.9 (6.8–9.0)	7.9 (7.1–8.7)	0.81
Time from ED to OR (h:mm)	1:03 (0:39–2:55)	1:15 (0:55–2:27)	0.07
Duration of surgery (h:mm)	2:05 (1:20–2:50)	2:00 (1:30–3:00)	0.54
MODS	25 (18)	26 (15)	0.41
ARDS	6 (4)	6 (3)	0.66
Infectious complications	63 (46)	70 (44)	0.69
Thrombo-embolic complications	15 (11)	19 (11)	0.97
Mortality	29 (21)	30 (17)	0.36

Data are expressed in median (IQR) or absolute numbers (%)

*MOI* mechanism of injury, *ISS* injury severity score, *SBP* systolic blood pressure, *Hb* hemoglobin, *BD* base deficit, *ED* emergency department, *OR* operating room, *MODS* multiple organ dysfunction syndrome

**Fig. 6:**
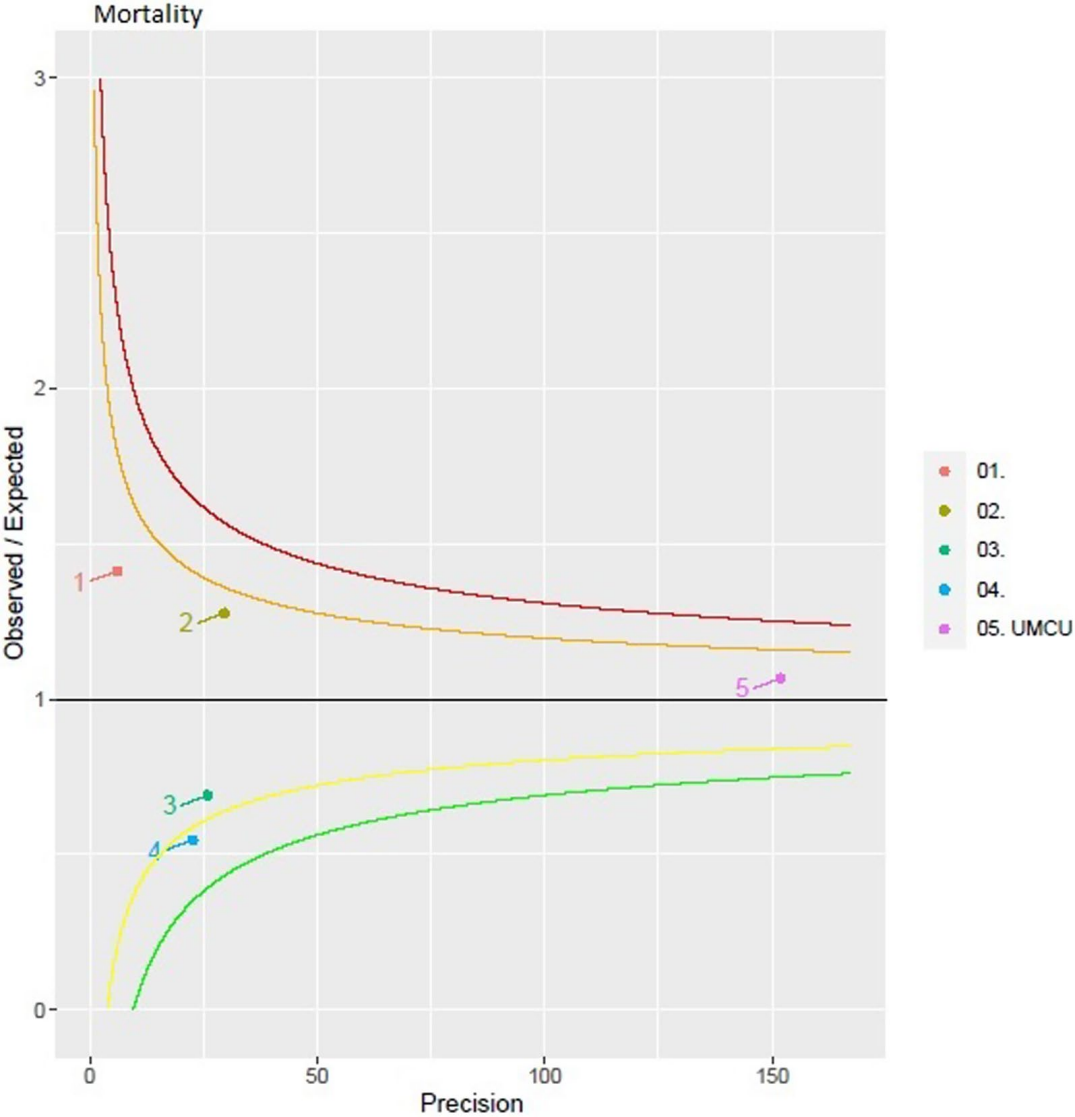
Standardized Mortality Ratio (SMR) in 2021. Numbers 01 through 04 are level 2/3 trauma centers within the region. Level-1 trauma center UMCU is depicted as number 05. Red and green lines represent 99.8% confidence intervals (CI), orange and yellow lines 95% CI. The funnel plot is provided by the Dutch National Trauma Registry

 Figure 6 shows a funnel plot with the survival mortality ratio (SMR) for the central region of the Netherlands in 2021. Numbers 01 through 04 represent the level 2/3 trauma centers within the region. Number 05 representing Level-1 trauma center UMCU shows a SMR of 1.07 suggesting that observed mortality was comparable to the expected mortality. SMRs for 2018 throughout 2020 were also approximately 1 (1.01, 1.08, and 1.06 respectively, Additional file 1: Figure S1).

## Discussion

In this study of severely injured patients in need of urgent surgery and ICU support who were cared for in a setting of a 24/7 available double trauma surgeon coverage, it was demonstrated that accuracy in (surgical) decision making was high based on adequate sequelae and appropriate use of damage control indication, with overall short transfer times. This was independent of time of day. Preventable death rate was low (< 2%). Over the years, the observed mortality was similar to the expected mortality suggesting good performance.

Nineteen percent of patients died with TBI (68%) as the most common cause of death. Exsanguination rate was 8%, and death by MODS even lower (3%). In a previous study we have shown similar exsanguination and MODS rates [19, 20] which were lower compared to other studies [21, 22]. Twenty-one percent of patients who underwent a laparotomy died, which is comparable to a large multi-center American study [22]. However, cause of death was different; in this study 4% of patients who underwent an urgent laparotomy died due to hemorrhage compared to 60% in the American study, which might at least partially reflect the differences in blunt and penetrating trauma, and prehospital distance to a major trauma center. The low mortality rates in exsanguination and MODS have previously been described in several studies in which the trauma care was evaluated [3, 19, 20, 23]. This improvement in trauma care in the last decades is likely caused by a combination of advances in prehospital treatment, hemostatic resuscitation, and damage control surgery. A dedicated trauma team such as a double on-call trauma surgeon service ensures continuous high quality trauma care in a high volume major trauma center, which was key to achieve these outcomes [23–25].

In-hospital transport times showed that treatment in ED was less than 32 min irrespective of the next destination (either CT or OR depending on physiology and type of injuries). Time in ED was shorter compared to other studies in which time from ED to OR for urgent laparotomy ranged from 42 to 55 min [22, 26, 27]. Additionally, time in OR was approximately 2 h including anesthesia (for both DCS and EDC), demonstrating not only that the surgical decision making was efficient, but also that OR time was efficiently used.

Many Level-1 trauma centers have a CT scan located in ED nowadays. Even though an immediate CT scan has not demonstrated to decrease in-hospital mortality nor to reduce time to bleeding control [28–30], it does facilitate logistics in patient transfer and decreases time to imaging. Despite the fact that, during the study period, patients needed to be transported to the CT-scan which was located in the radiology department, median transport times from ED to CT-scan were comparable (32 min) to ED-CT times in patients in the REACT trial who had a total-body CT with the CT-scanner located in ED [30]. This suggests that time in ED in our study was relatively short and that transport from ED to CT-scan was fast.

In this study there was no difference in mortality rate between DCS patients and EDC patients even though DCS patients were more acidotic and in need of more blood products. Further, DCS patients were more quickly in OR with shorter duration of surgery. This demonstrated that, although retrospective analysis was performed, the selection of patients who needed damage control surgery was accurate.

To our knowledge, this is the first report in which outcome of severely injured patients was described in a Level-1 trauma center with a unique on-call system with a 24/7 double trauma surgeon on-call service. A frequently noted disadvantage of such a system that is that the on-call frequency is high. However, when asked, all trauma surgeons in our center accepted high on-call frequencies in exchange for an extra set of experienced hands during surgery. This not only facilitates the (more complex) surgical procedures, but also reduces time in OR. In a period of ongoing specialisation, work hour regulations, and decreased exposure in OR, this system also facilitates young, less experienced trauma surgeons to fully participate in treating severely injured patients with associated complex decision making since there is always another trauma surgeon to help. Further, a double trauma surgeon on-call system also decreases the moral distress caused by the fact that a single surgeon on-call held in OR to help one patient is in fear of not being able to help the next patient who needs urgent care. With annually 375 severely injured patients this is a frequently recurrent issue. In the era of burn-out prevention, this moral distress should not be ignored and receive attention since it is a major issue in Level-1 trauma centers with junior residents on-call who need direct supervision in treating the severely injured patient [31]. Although no data were collected on the frequency of the necessity for the second surgeon’s presence in ED, an estimation could be calculated; Fifty-six percent of patients in our study had urgent surgery after office hours which means that during surgery the second surgeon is also present in the hospital. Further, 49% of the 1400 patients (375 of them severely injured) who annually present in ED with trauma team activation arrive between 17.00 and 08.00 [6]. This means that, on average almost 4 patients (one of whom is severely injured) arrive in ED on a daily basis, half the time after hours. As a consequence, the second surgeon needs to leave OR to be present in ED to lead the resuscitation. These calculations are likely even underestimated since non-severely injured patients with for example open fractures who also need urgent surgery were not included, nor were weekend days calculated separately.

There are several limitation in this study. One of the limitations of this study is that it was conducted in a single institution with a predominantly blunt trauma population in which the clinical treatment and research were conducted by the same clinicians. Even though the studied cohort is a unique trauma population we feel it is representative for urban areas with short pre-hospital transport times and predominantly blunt trauma. In this study it was decided to only include patients who needed urgent surgery and ICU support, since decision making in this population is most important and urgent. However, swift transport times and accuracy in decision making is equally important in all patients in need of urgent care. Since it was demonstrated that decision making was accurate in the most severely injured patient population it could be assumed this would also be true for all patients.

## Conclusions

In this cohort of severely injured patients in need of urgent surgery and ICU support it was demonstrated that surgical decision making was swift and accurate with low preventable death rates. 24/7 Physical presence of a dedicated trauma team has likely contributed to these good outcomes.

### Supplementary Information

**Additional file 1**. **Figure S1**. **A**. Standardized Mortality Ratio in 2018. **B**. Standardized Mortality Ratio in 2019. **C**. Standardized Mortality Ratio in 2020.

**Additional file 2**. **Table S1**. Damage control surgery (DCS) and early definite care (EDC) in deceased patients.

## Data Availability

The dataset supporting the conclusions of this article are available upon reasonable request from the corresponding author.

## Abbreviations

AIS: Abbreviated injury scale
ARDS: Adult respiratory distress syndrome
CNS: Central nervous system
DCS: Damage control surgery
DNTR: Dutch national trauma registry
ED: Emergency department
EDC: Early definitive care
FFP: Fresh frozen plasma
GCS: Glasgow coma scale
ICU: Intensive Care Unit
IQR: Interquartile range
ISS: Injury severity score
MODS: Multiple organ dysfunction syndrome
MOF: Multiple organ failure
OR: Operating room
PLT: Platelets
PRBC: Packed red blood cells
SMR: Standardized mortality ratio
SOFA: Sequential organ failure assessment
TRISS: Trauma Injury Severity Score
