# Supplementary material for: Outcome of severely injured patients in a unique trauma system with 24/7 double trauma surgeon on-call service
Source: Scand J Trauma Resusc Emerg Med. 2023 Oct 25;31:60. doi: 10.1186/s13049-023-01122-9 (PMC10598943; doi:10.1186/s13049-023-01122-9)
Supplement: Supplementary file 1 — Additional file 1. Figure S1. A. Standardized Mortality Ratio in 2018. B. Standardized Mortality Ratio in 2019. C. Standardized Mortality Ratio in 2020. [file 13049_2023_1122_MOESM1_ESM.docx]

**Figure S1.**

1. Standardized Mortality Ratio in 2018


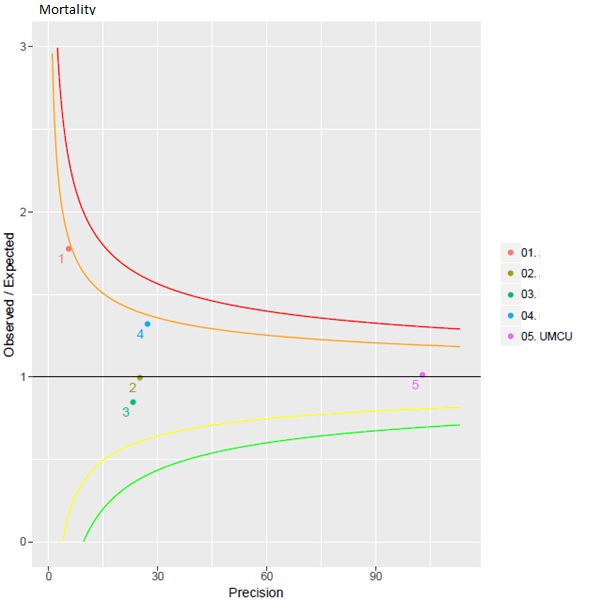


1. Standardized Mortality Ratio in 2019


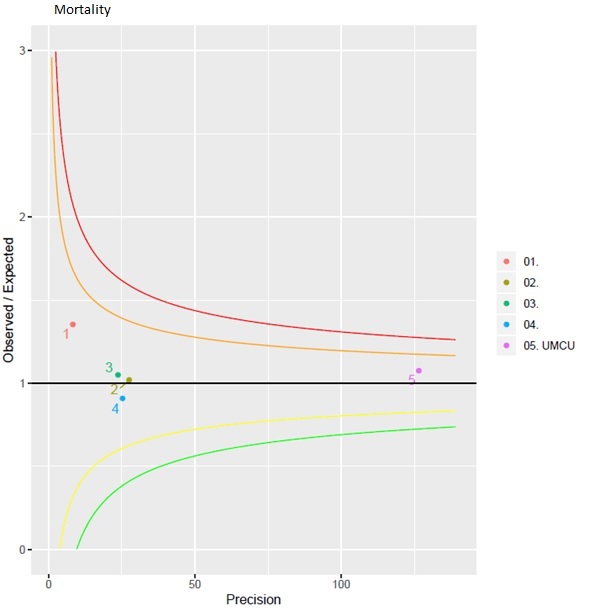


1. Standardized Mortality Ratio in 2020


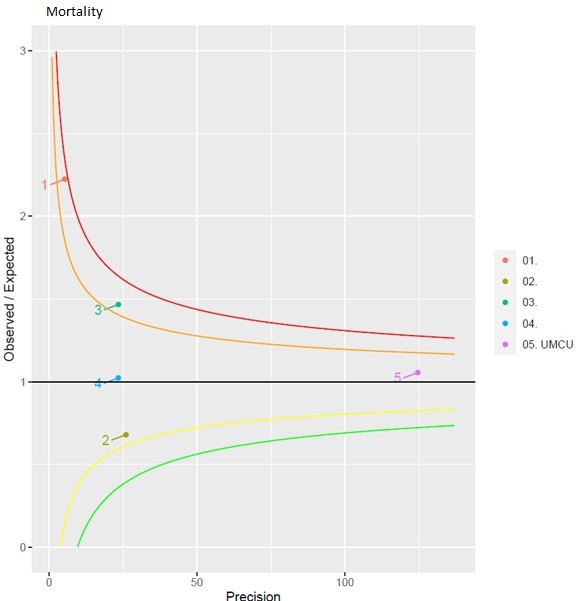


Numbers 01 through 04 are level 2/3 trauma centers within the region. Level 1 trauma center UMCU is depicted as number 05.

Red and green lines are 99.8% confidence intervals (CI), orange and yellow lines are 95% CI.

The funnel plots are provided by the Dutch National Trauma Registry.
