# Supplementary material for: Outcome of severely injured patients in a unique trauma system with 24/7 double trauma surgeon on-call service
Source: Scand J Trauma Resusc Emerg Med. 2023 Oct 25;31:60. doi: 10.1186/s13049-023-01122-9 (PMC10598943; doi:10.1186/s13049-023-01122-9)
Supplement: Supplementary file 2 — Additional file 2. Table S1. Damage control surgery (DCS) and early definite care (EDC) in deceased patients. [file 13049_2023_1122_MOESM2_ESM.docx]

**Table S1.** Damage control surgery (DCS) and early definite care (EDC) in deceased patients.

| OR-1 | DCS  (n=28) | EDC  (n=31) | P-value |
| --- | --- | --- | --- |
| Age (years) | 52 (32-72) | 69 (45-73) | 0.18 |
| ISS | 41 (30-50) | 38 (29-43) | 0.05 |
| AIS head | 3 (0-4) | 4 (4-5) | 0.004* |
| AIS face | 0 (0-1) | 0 (0-2) | 0.02* |
| AIS chest | 4 (3-4) | 3 (2-4) | 0.02* |
| AIS abdomen | 4 (2-4) | 0 (0-2) | <0.001* |
| AIS pelvis/extremities | 3 (2-3) | 2 (2-3) | 0.08 |
| AIS external | 0 (0-1) | 0 (0-1) | 0.58 |
| Time from ED to OR (h:mm) | 0:57 (0:28-1:29) | 1:12 (0:51-3:49) | 0.01* |
| OR Duration (h:mm) | 2:00 (1:21-2:52) | 2:00 (1:20-3:00) | 1.0 |
| BD_OR (mEq/L) | -12.0 (-20.5—7.0) | -5.0 (-8.0—2.0) | <0.001* |
| Hb_OR (mmol/L) | 6.4 (5.2-7.2) | 6.3 (5.5-7.8) | 0.68 |
| Temperature_OR (^o^C) | 33.7 (32.9-35.3) | 34.7 (33.6-35.3) | 0.32 |
| Crystalloids_OR (L) | 3.0 (2.0-3.8) | 4.0 (2.0-5.0) | 0.11 |
| PRBC_OR (U) | 8 (3-15) | 1 (0-3) | <0.001* |
| FFP_OR (U) | 8 (4-15) | 0 (0-3) | <0.001* |
| PLT_OR (U) ^#^ | 2 (0-3) | 0 (0-1) | 0.001* |
| Surgical procedure** |  |  | **Total** |
| Thoracotomy | 3 (8) | 1 (3) | 4 (6) |
| Laparotomy | 21 (53) | 3 (9) | 24 (33) |
| Craniotomy | 2 (5) | 12 (38) | 14 (19) |
| Spine fixation | 0 | 3 (9) | 3 (4) |
| Fracture fixation | 1 (3) | 1 (3) | 2 (3) |
| External fixator extremities/pelvis | 12 (30) | 0 | 12 (17) |
| Vascular procedure | 1 (3) | 1 (3) | 2 (3) |
| Miscellaneous^§^ | 0 | 11 (34) | 11 (15) |
| Total | 40 | 32 | 72 |

Data are expressed in median (IQR) or absolute numbers (%), * statistically significant

^#^ 1 unit of platelets contains 5 donors

**several patients had more than one surgical procedure

^§^ miscellaneous procedures included insertion of ICP meter, extraventricular drain, haloframe, amputation extremity, fasciotomy, debridement of soft tissue injuries, neck exploration

ISS=injury severity score, AIS=abbreviated injury scale, ED=Emergency Department, OR=operating room, Hb=hemoglobin, BD=Base Deficit, PRBC=packed red blood cells, FFP=fresh frozen plasma, PLT=platelets
